# The energy challenges of artificial superintelligence

**DOI:** 10.3389/frai.2023.1240653

**Published:** 2023-10-24

**Authors:** Klaus M. Stiefel, Jay S. Coggan

**Affiliations:** NeuroLinx Research Institute, La Jolla, CA, United States

**Keywords:** brain energy, bioflop, bioalgorithm, biomimicry, artificial general intelligence, thermodynamics of computation, artificial intelligence

## Abstract

We argue here that contemporary semiconductor computing technology poses a significant if not insurmountable barrier to the emergence of any artificial general intelligence system, let alone one anticipated by many to be “superintelligent”. This limit on artificial superintelligence (ASI) emerges from the energy requirements of a system that would be more intelligent but orders of magnitude less efficient in energy use than human brains. An ASI would have to supersede not only a single brain but a large population given the effects of collective behavior on the advancement of societies, further multiplying the energy requirement. A hypothetical ASI would likely consume orders of magnitude more energy than what is available in highly-industrialized nations. We estimate the energy use of ASI with an equation we term the “Erasi equation”, for the *E*nergy *R*equirement for *A*rtificial *S*uper*I*ntelligence. Additional efficiency consequences will emerge from the current unfocussed and scattered developmental trajectory of AI research. Taken together, these arguments suggest that the emergence of an ASI is highly unlikely in the foreseeable future based on current computer architectures, primarily due to energy constraints, with biomimicry or other new technologies being possible solutions.

## Introduction

The possible emergence of an artificial superintelligence (ASI) has been the subject of much academic discussion (Carlsmith, 2022). The idea of an entity which is significantly smarter than humans, comparable perhaps to the difference between humans and great apes, captures the human imagination. So much different is human society than chimpanzee society, for example, that one could imagine an ASI easily tackling some of the most daunting problems facing humanity such as solving gravity, ecosystem management, space travel and, not ironically, sustainable and affordable energy. Moreover, much as the next smartest primates cannot begin to understand human technology, it is fun to speculate about what an ASI could come up with that would leave us equally gobsmacked.

Science fiction literature has not surprisingly also had it's say, with Lem coining the term “intellelectronics” (Lem, 1964). Here, we outline arguments that such a superintelligence is unlikely to be realized any time soon with current technology due to its projected energy requirements. An important point in this context is the definition of an ASI. It is difficult to precisely define an entity which doesn't exist (yet), but its eventual architecture is neither known nor relevant for the present discussion, as the main argument relates to the estimated minimum energy use of such a system, which is independent of technical details, in the same way that a car is different from an ox, but the work (energy) needed to pull a cart a given distance is the same in both cases.

We want to clarify from the start that we understand the definition of intelligence to be contentious and multifaceted and this is no less true of the concept of intelligent computing (e.g., Zhu et al., 2023). But no matter the definition, strategy, technique or algorithmic approach (e.g., Hochreiter and Schmidhuber, 1997; Hinton et al., 2006), it is widely agreed that the basis involves fundamental computations and to develop a narrative on ASI and its costs, we consider intelligence to be the product of a very large number of computations, performed or emergent from the dynamics of biological tissue or manufactured information processors such as semiconductor chips. We posit that equivalence in intelligence is only possible when the same magnitude of complexity of computations per time is executed with comparable and intelligible outputs. We don't consider “shortcuts” via 20,000 lines (just an example) of very clever program code to be solutions.

This reasoning excludes successes of AI in limited domains, like maze navigation or written text production, as proofs of machine intelligence equivalent to human. Just because a robot is as good as a human in navigating a maze or even faster at recombining training data does not make it as intelligent. Equivalent intelligence will only be achieved when the highest human cognitive abilities are replicated, including those requiring agency and adaption, and a superintelligence will need to surpass these in competence at least and probably in speed as well. But however one defines intelligence, our presumption is that it will not be achieved without equivalent computational complexity.

The issue of what exactly the hypothetical ASI does, whether it is directly in control of effectors (for instance the power grid of countries) or acts as an “advisor” for a government or private entity, is not relevant. The definition we use encompasses any man-made computational system significantly more intelligent than humans, not only with the capability to solve problems we cannot, but possibly with the ability to control the human population by means of manipulation, superior planning, or direct force if incorporated into robots.

## Results

The main argument that indicates that the emergence of an ASI is highly unlikely in the foreseeable future rests on the fact that the energetic cost of the computations performed would by far surpass the energy supply available to human civilization. While we believe that ASI is technologically impossible to implement in present-day semiconductor technology and its high energy use, we do not believe that it is impossible in principle, as other authors do (Roli et al., 2022).

### Energy use in biological and engineered computation

Whatever the architecture of an ASI turns out to be, it will be bound by the principles of thermodynamics of computation (Bennett, 1982). Reversible computation with no dissipation of energy has been proposed to work in principle (Frank, 2005), but is unlikely to be possible on the speeds necessary for conventional processors or even an ASI system, with great numbers of individual operations needing to be performed at great speeds.

A human brain contains about 10^11^ neurons and consumes about 12 W. A typical laptop processor uses 150 W. The fastest supercomputer at the time of this writing, Frontier, uses 21 × 10^6^ W to perform 1.685 ExaFLOPS (1.685 × 10^18^ floating point operations per second). Assigning a computational speed to nervous systems commensurable to the widely used unit of computational power for digital computers, floating point operations per second (FLOPS), is at least not trivial, or at worst a mismeasurement or simply not comparable.

We hence give an order-of magnitude estimate of the computational efficiency of present-day semiconductor processors executing AI algorithms in comparison to biological brains (Schuman et al., 2022). To do this we compare the energy use of a state-of-the-art, detailed simulation of parts of a mammalian brain to the energy use of an actual brain.

Our example comes from Switzerland's Blue Brain Project (BBP) of EPFL, which has been creating a biologically realistic, data-driven reconstruction and simulation of an entire mouse brain.^1^ This intricate simulation includes details of molecules, cells, circuits and brain regions that together participate in biological computation (e.g., Markram et al., 2015; Ramaswamy et al., 2018; Reimann et al., 2019; Zisis et al., 2021; Coggan et al., 2022).

The BBP uses a supercomputer roughly capable of 2 × 10^3^ TFLOPS, with 400 TB of memory and 200 TB/s of memory bandwidth. The energy use for 720 processors involved in this simulation is around 400 kW. A simulation of 10 million neurons in a cortical circuit requires approximately 1,460 TFLOPS and 270 kW to simulate 1 s of biological time and took more than 8 h of processing time, slower than nature by about a factor of 3 × 10^4^. If we convert power (W or J/s) to energy (J) units, 270 kW (for 8 h) is 7,776,000,000 J of energy to compute 1 s of mouse cortical activity.

**Figure 1:**
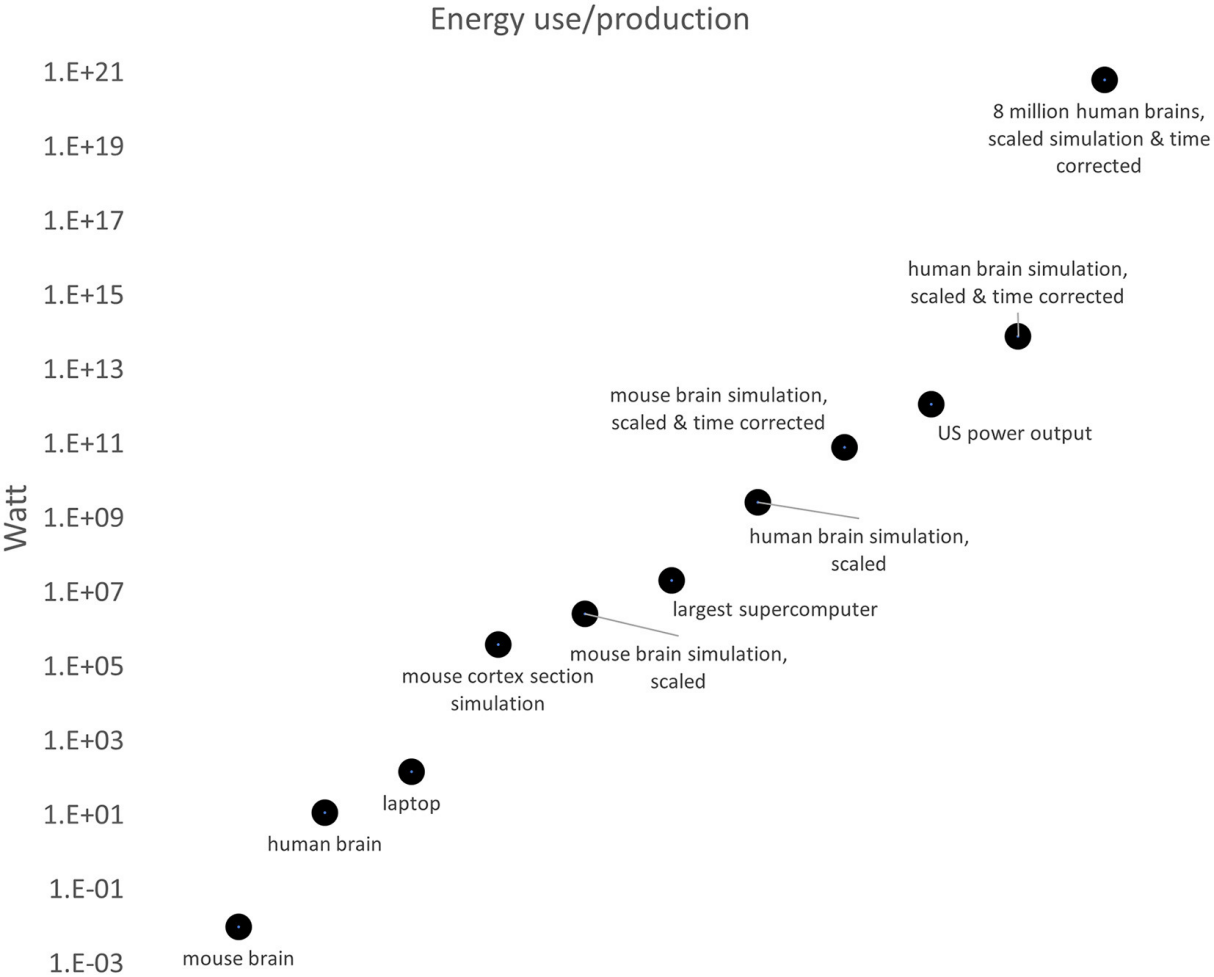
Energy use by the brain of a mouse, a human, a typical laptop processor, a leading supercomputer (Frontier), and the scaled energy uses (with and without corrections for processing time) for a complete mouse brain, a complete human brain and 8 million human brains.

When extrapolating to the entire mouse brain with 10^8^ neurons, a simulation would require 2.7 MW. Extrapolating again to a human brain with 10^3^ times as many neurons as a mouse brain, the power requirement would be 2.7 GW (which is 7.7 × 10^13^ J for 1 second of thought and 14.6 ExaFLOPS). This is orders of magnitude above the amount of energy a human biological brain is estimated to use, at 12 W. Based on the detailed simulations conducted by the BBP example, we estimate that biological computing is about 9 × 10^8^ times more energy efficient than artificial computing architecture (Figure 1).

We stress that this estimate is a lower bound. Although the simulations of the BBP are already highly detailed and the simulation is continuously increasing its biologically realistic complexity, the current energy estimates for simulations are a snapshot and do not yet take into consideration a significant amount of the computational complexity of brains. For example, many information-bearing processes of single cells are yet to be incorporated, both in gray and white matter (not to mention glia), such as local dendritic integration, allosteric proteins, biomolecular networks, spatial integration, neuromodulatory elements, synaptic plasticity, gap junctions and ephaptic effects. Adaptation and learning factors will also require computational resources. In addition, for the fundamental energy costs of computation in biological brains, and in comparison to artificial information processing networks, we have to subtract the costs of creating and maintaining the infrastructure. Even with some uncertainty about how these costs are distributed and assuming some overlap, it is clear that, in the example of the human brain, the actual cost of computation is actually much lower and the 12 Watts measured. For all of these reasons, the estimated 9 × 10^8^ times energy efficiency differential for a large BBP mouse brain simulation still grossly underestimates the true value.

### Computing time considerations

This estimate above is based on 1 s of simulated biological time, but considering that it takes 3 × 10^4^ times longer for the BBP supercomputer to simulate biological time, these simulations cannot be considered equivalent. Performing an action 30,000 times slower is necessarily less energy demanding. The most straightforward way to correct for this discrepancy is to multiply the relative energy efficiency of 9 × 10^8^, derived above, by the 3 × 10^4^, and we arrive at 2.7 × 10^13^ as the total relative efficiency of the human brain vs. silicone semiconductor processors running AI algorithms.

### Simulation vs. emulation

The above approach is relevant especially since neuromorphic computing, computing based on architectures inspired by brain structure and function, is increasingly seen as a preferred strategy for implementing efficient computations (Indiveri et al., 2011; Wang et al., 2013; Schuman et al., 2022). However, an important argument is that in order to replicate the performance of a human brain, one does not have to reproduce the exact structure and function of its biological intricacies. We agree with this notion, but argue that in any case the same amount of computation has to be carried out.

**Figure 2:**
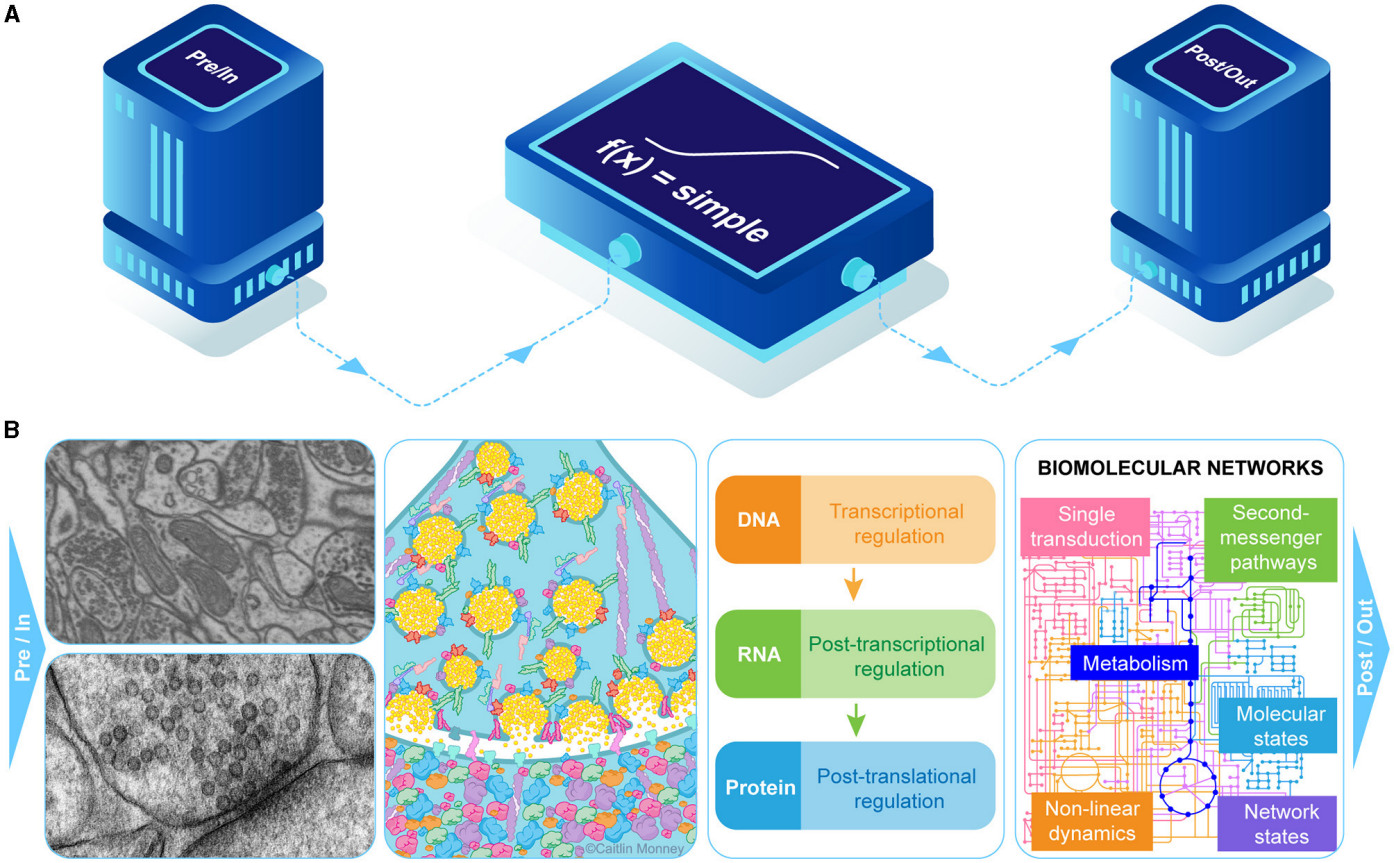
Juxtaposition of a highly simplified “synapse” as commonly used in a large-scale brain simulation with some of the details (not comprehensive list) of a biological synapse. **(A)** diagram of a typical computational representation of information flow and processing from a presynaptic or pre-point neuron input source (pre/in) through a simple transformation function [f(x) = simple] to an output or postsynaptic state (post/out). **(B)** left panel, top: shown are a small section of dense neuropil along with pre- and postsynaptic structure (left panel bottom) in an electron micrograph; second panel: some of the multi-protein complexes involved in vesicle docking and postsynaptic reception as in the NMDA-type glutamate receptor, structures involved in computation; third panel: regulation of transcription and translation affect cell's computational state and capabilities; panel 4: pathways in many biomolecular networks transduce, process and store information about cell state and affect information throughput.

Without doubt, a single neuron is capable of complex computations, and while they don't have to be simulated as electrical potentials traveling along axons and dendrites, the input/output relationships will have to be similarly complex (e.g., Attwell and Laughlin, 2001; Gidon et al., 2020). Highly simplified analog sigmoid transfer-function model “neurons” (often referred to as “point neurons”) with highly simplified “synapses” will certainly not suffice. Beyond the biophysical and electrical features of neurons based on their complements of ion channels and neuromorphology, there are many other layers of information processing involving modifications of the cell's internal states including macromolecular shape changes and rate functions, genetic, transcriptional, translational, epigenetic, biomolecular networks, second messenger pathways and energy distributions that affect neuronal output (Figure 2; Ananthanarayanan et al., 2009; Eliasmith and Trujillo, 2014).

A human-brain like intelligence will not likely emerge from short-cut simulations of a human brain. Rather, such an intelligence (or greater) will most likely emerge from a device with a similar order of magnitude of complexity. An emulation of a human brain is unlikely to succeed if built with highly simplified components arranged in a massively simpler way than biological brains are arranged. And even an estimated improvement of energy efficiency by a factor of 10^3^ by an emulation (without precise biological detail) vs. a simulation will only reduce, but not solve, the fundamental energetic problems outlined above. It seems completely improbable, on energetic grounds, to surpass biological brains when using silicone semiconductor processors.

We speculate that only an approach that closely resembles biological computing strategies will be able to compete with biological intelligence. For example, an alternative set of large organic molecules, arranged in a multi-scale system, might be made to compute as efficiently as a brain. While there is probably no necessity to use proteins and nucleic acids *per se* to build cells, the principles of neuroscience or general single cell biology will have to be followed to be as energy efficient as biology (e.g., Hassabis et al., 2017). The pursuit of ASI might well benefit from biomimicry beyond today's neuromorphic strategies.

### Human group intelligence

Humans are inherently social animals, it is therefore reasonable to compare the energy use of the brains of large human populations with that of a proposed ASI. We, therefore, have to assign the energy use of 8 billion human brains (estimated global population in 2022) to the human “group intelligence”. Although each human brain contributes a different task to the performance of the species, it is the sum total that drives the species forward with the intelligence required for group survival (or extinction should our intelligence falter). In reality, even the tasks performed in the construction of a footpath (involving spatial planning and the use of several tools to manipulate a variety of materials) require greater computational performance than any advanced AI system can do in 2023.

It is already remarkable that even given the astonishing computational efficiency of brains compared to computers, a large part of the planetary land area has already been modified to feed humans, and a large part of the caloric intake of humans is metabolically used by their brains (10× greater/mass than other tissues). This measure will not scale linearly, and the cognitive output of a collaborative group of 10 humans will not equal 10 times the output of a single human. Rather than trying to determine a precise multiplicative factor, we want to include a rough estimate of the cognitive ability by collaborative groups of humans into our estimate. Human groups are far superior than individual humans in terms of problem-solving (persistent isolation of humans even leads to severe psychological problems, although we are not sure this would be true of ASI).

### Improvement in understanding reality

Another important point is by how much ASI will have to outperform humans. An often cited analogy is that ASI will be relative to humans, as we are relative to great apes. The brain of a chimpanzee is about a third the size of a human brain. Expecting one-third of the computational power and corresponding energy use for chimps is probably a reasonable minimum assumption. Taken together, a hypothetical ASI will have to outcompete the collective intelligence of eight billion humans, each with highly energy efficient brains, and it will likely have to outcompete them by a margin of at least three.

### ASI energy demand

To outcompete human collective intelligence within the present technological boundaries by a large margin, an ASI would have to consume a considerable amount of energy. The equation describing this energy use is:


(1) $$\begin{array}{l} E_{ASI}=E_{brain}fGs \end{array}$$

*Energy use for ASI (E*_*ASI*_*)* = *Energy use per brain (E*_*brain*_*) X relative computational efficiency brain/AI (f) X human group intelligence group size (G) X AI superiority (s). E*_*brain*_ and *E*_*ASI*_ are in Watts, all other parameters are unit-less. We name this equation the **Erasi Equation** (**E**nergy **R**equirement of **A**rtificial **S**uper**I**ntelligence).

The best assumptions which we derive here are that the relative efficiency is 2.7 × 10^13^ times worse in computer hardware (a measure derived from detailed brain simulations, see above), and that we need to compare the performance of an ASI to the combined intellectual output of 8 × 10^9^ humans. Additionally, the assumption is that an ASI would have to supersede human intelligence by a factor of 3, derived from the human-chimpanzee difference. In this case the following calculation represents our best guess for the cost of ASI:


(2) $$\begin{array}{l} E_{ASI}=\left(12W\right)\left(2.7\;\times \;10^{13}\right)\left(8\;\times \;10^{9}\right)\left(3\right)=7.78\;\times \;10^{24}W \end{array}$$

An alternative, much more optimistic assumption might be that ASI would have to supersede the capacity of only a single human brain. In this case the energy use would be:


(3) $$\begin{array}{l} E_{ASI}=\left(12W\right)\left(2.7\;\times \;10^{13}\right)\left(1\right)\left(3\right)=9.7\;\times \;10^{14}W \end{array}$$

In February 2022, the US had a power generation capacity of more than 1.2 × 10^6^ MW (1.2 × 10^12^ W). Hence the ASI would consume power somewhere between one thousand and one trillion times larger than the power generation of the USA, an obviously unrealistically high range, and a situation that precludes the emergence of an ASI in the absence of radical engineering advances.

Just like in the case of the Drake equation (Wallenhorst, 1981), the equation describing the number of likely technological civilizations in the galaxy, the Erasi equation describes the energy requirement for ASI given a set of assumptions. Just as in the Drake equation, the assumptions are up for discussion, and values for revised assumptions can be plugged-in. We argue that with any reasonable set of assumptions, the energy use will be orders of magnitude higher than that of a large, highly industrialized nation.

## Discussion

The intellectual and political discourse of the future of AI has recently focused on the potential dangers of an “AI takeover” by an ASI. Here we argue that the basic thermodynamics of computation make such a takeover highly unlikely anytime soon and probably never without significant changes in the physics of computation.

AI has brought impressive results and multiple practical uses which have already changed society. But despite these successes, our arguments demonstrate, in isolation and synergistically with each other, that it is highly unlikely, if not impossible, for an ASI to emerge which will turn humans into slaves. It is likewise premature to expect salvation from ASI-like architectures in the form of the hypothesized “singularity”, a word coined by the physicist John von Neumann, and meaning a time when people could upload their virtual brains into an eternal cyber-world, thus achieving immortality.

While we propose that an ASI is unlikely on energetic grounds, we disagree with arguments like those in Roli et al. (2022) that only biological organisms can show agency and hence no non-biological entity can achieve a high level of cognitive functioning. That said, biomimicry has proven to be a very effective way of making scientific and engineering progress. Nature has already solved many of the problems we struggle with today, if only we would take note. We must re-double our efforts to discover what is effectively a “bioflop” and learn it's principles in order to engineer a manageable equivalent. The bioflop will likely involve continuous or analog information processing. Likewise, beyond the flop there is the algorithm and identifying bioalgorithms will be equally important and likely involve multiple scales of information processing. We think the current AI algorithmic approaches are uni-scale and thus are likely missing some of the major points of evolved biological computing.

In essence, we believe that the intricate multi-level architecture of biological brains makes them so much more energy-efficient at computing that they can achieve computational powers far beyond what is possible with silicone-based semiconductor chips. We might only be able to build energy efficient AGI with organic molecules following the same rules as in biology (perhaps rediscovering ourselves in the process). Some form of synthetic biology to emulate the energy efficiency of biology will likely be required. Such breakthroughs could come from the new field of organoid intelligence or one of it's spin-offs (Kagan et al., 2022; Smirnova et al., 2023). The whole approach of using microchips is likely doomed to fail in this task, we will need a revolutionary understanding of information processing and how to achieve it with molecules arranged in multiple levels in order to achieve ASI.

Despite the success of smart chatbots such as ChatGPT (OpenAI, 2022) and the ever growing slew of clever large language algorithms that combine training data to produce a mostly cogent interface for the prompted distillation of information, doubts persist. While chatbots do pose some interesting challenges to concepts in cognitive linguistics or semantics, the question of true understanding remains (Raikov, 2021). Furthermore, intelligent or not, even OpenAI's Sam Altman recently stated that the computational costs of ChatGPT were “eye-watering”^2^ and the full reckoning of the thermodynamic impact of LLM's has yet to be even estimated.

We propose that to achieve a comparable amount of computation as a human brain, a comparable amount of complexity is necessary, independently of how this complexity is brought about (via a biological brain or in a completely different, but comparably complex machine). No clever codes will produce the same output as a human brain does because clever algorithms are neither robust, flexible nor adaptable and therefore not truly intelligent. The proposal of our ERASI equation is not intended to be the final but rather the initialization of a conversation about the costs of computation, both natural and artificial. The broader AI and biology research communities are encouraged to add their voices or equation term suggestions to this dialog.

Among the prospective computing technologies that have been cited as significantly more energy efficient are quantum computing and optical computing. We believe that our arguments are not well countered by technologies which are not yet reliably working on a large, relevant scale, but we nevertheless would like to address their potentials.

In quantum computing, the state of the art (as of 2017) is the 2,000-Qbits quantum annealer manufactured by D-Wave Systems. The system is faster than any existing supercomputer approximately by a factor of 3,600. However, with a capacity of 2,000 bits, it is still highly limited, especially since the machine processing 2,000 bits is sized 3 × 2 × 3 meters (Elsayed et al., 2019). We do not consider quantum computing a solution to the conundrum we point out in this paper in the foreseeable future.

Optical computing is much further ahead in terms of practical development, and based on the current state of the technology, concrete quantitative predictions promise that photonic CMOS will eventually enable an energy efficiency of 0.3 picoJoules/bit with 16 nm CMOS (Young et al., 2010). These are highly significant improvements, but two caveats remain: the optical technology will greatly improve data transmission, but not computation itself, and more importantly, given the order-of-magnitude discrepancy between needed and available (with present day computing technology), even a significant improvement of energy efficiency as that promised by optical computing will not solve the problem we outline.

### Additional science policy arguments

Not only is the emergence of an ASI unlikely for energetic reasons, but it is also not the path which the majority of research into AI is taking presently. This is both true for the commercial applications of AI as in academic research. The majority of research in AI appears to be concerned with classification and sorting tasks, as well as with autonomous spatial navigation. By any standards these efforts are very successful, including success in classification tasks in very high dimensional data spaces. The very successful approach of deep learning is a specialized engineering solution for classifying such high-dimensional data (Sejnowski, 2018).

AI has produced extremely impressive results in limited domains which are very dissimilar from what humans have evolved to do. One example is the success in chess, where the reigning world champion was first defeated by software in 1997. It can be argued that in chess, AI has reached superhuman intelligence. However, the intellectual challenges in chess, a highly formalized game of logic, are very different from those encountered in navigating and manipulating the real world.

Artificial general intelligence (AGI), potentially leading to an ASI, is a niche within research in AI and is not receiving the attention which many other subfields do. ASI will not likely emerge by chance, just as nuclear weapons, intercontinental ballistic missiles and particle colliders (to name three of many examples) did not emerge by chance from efforts in somewhat related disciplines, but were the results of massive, concentrated efforts of large numbers of scientists, engineers and support personal. The Blue Brain Project of EPFL in Switzerland provides a potential structural enterprise model for such an effort. The socio-political situation in AI research on its own does not preclude the development of ASI, but in the present day it acts in synergy with the argument about the energy consumption.

## Data availability statement

The original contributions presented in the study are included in the article/supplementary material, further inquiries can be directed to the corresponding author.

## Author contributions

KS and JC: conceptualization and writing—original draft. All authors contributed to the article and approved the submitted version.

## Footnotes

^1^ https://www.epfl.ch/research/domains/bluebrain/

^2^ https://techcrunch.com/2023/01/11/openai-begins-piloting-chatgpt-professional-a-premium-version-of-its-viral-chatbot/
